# High Performance Liquid Chromatography versus Stacking-Micellar Electrokinetic Chromatography for the Determination of Potentially Toxic Alkenylbenzenes in Food Flavouring Ingredients

**DOI:** 10.3390/molecules27010013

**Published:** 2021-12-21

**Authors:** Huynh N. P. Dang, Joselito P. Quirino

**Affiliations:** Australian Centre for Research on Separation Science (ACROSS), School of Natural Sciences—Chemistry, University of Tasmania, Hobart, TAS 7001, Australia; huynhngocphuong.dang@utas.edu.au

**Keywords:** alkenylbenzenes, food, high performance liquid chromatography, micellar electrokinetic chromatography

## Abstract

Alkenylbenzenes, including eugenol, methyleugenol, myristicin, safrole, and estragole, are potentially toxic phytochemicals, which are commonly found in foods. Occurrence data in foods depends on the quality of the analytical methodologies available. Here, we developed and compared modern reversed-phase high performance liquid chromatography (HPLC) and stacking-micellar electrokinetic chromatography (MEKC) methods for the determination of the above alkenylbenzenes in food flavouring ingredients. The analytical performance of HPLC was found better than the stacking-MEKC method. Compared to other HPLC methods found in the literature, our method was faster (total run time with conditioning of 15 min) and able to separate more alkenylbenzenes. In addition, the analytical methodology combining an optimized methanol extraction and proposed HPLC was then applied to actual food flavouring ingredients. This methodology should be applicable to actual food samples, and thus will be vital to future studies in the determination of alkenylbenzenes in food.

## 1. Introduction

The interest in alkenylbenzenes started in the 1960s, when safrole was found to cause tumours in rat liver [1]. Thereafter, other alkenylbenzenes have been studied to determine their hepatocarcinogenicity. In the early 2000s, the EU Scientific Committee on Food (EU-SCF) considered estragole, methyleugenol, and safrole as genotoxic and carcinogenic. Eugenol and myristicin are weak hepatocarcinogens, and the excessive consumption (e.g., >1 g) of myristicin can cause hallucinogenic effects due to its similar chemical structure to serotonin [2]. Thus, restrictions on the use of alkenylbenzenes as food additives have been recommended [3,4,5]. The EU maximum level is 1 mg/kg of safrole in foods and beverages. In the case of methyleugenol, estragole and other potentially toxic derivatives, the EU-SCF cannot establish their exposure limits in food [3,4,5]. Therefore, the margin of exposure approach had been used to assess the levels of some alkenylbenzenes in various food products [6,7,8].

There have been studies that indicated high levels of alkenylbenzenes in foods and beverages [9,10]. An issue with alkenylbenzenes is their widespread presence in many edible plants, which are typically used as spices/flavourings. Table 1 summarises the natural occurrence of alkenylbenzenes in this study (i.e., eugenol, methyleugenol, myristicin, safrole, and estragole) present in various edible plants/spices. A combination of the plants listed in Table 1 could potentially lead to high levels of the selected alkenylbenzenes in some foods.

Reliable analytical methods are extremely important in determining the occurrence of alkenylbenzenes in food. The data from these methods are used to assess the safety of food products and to calculate the total dietary intake. During the last decade, reported analytical methods for the determination of alkenylbenzenes in food and beverage samples have been mostly based on high-performance liquid chromatography (HPLC) [33]. The hydrophobic alkenylbenzenes were typically separated using a reversed-phase HPLC column and a mobile phase that contained an organic solvent. The analytes separate via their differential retention characteristics as they pass through the column that is pumped with a mobile phase. An alternative analytical separation technique to HPLC for alkenylbenzenes is micellar electrokinetic chromatography (MEKC) [34,35], which is a mode of capillary electrophoresis. Huhn and co-workers employed MEKC with negatively charged sodium dodecyl sulfate (SDS) micelles to separate the electrically neutral eugenol, safrole, methyleugenol, and myristicin in sassafras essential oil samples [36]. In MEKC, the micelles that were formed from SDS above the critical micelle concentration (CMC) acted as the chromatographic pseudophase. The analytes separate in the presence of an electric field, due to the differential migration of the alkenylbenzenes caused by their different affinities to the SDS micelles. MEKC is a greener technique compared to HPLC, but it suffers from poor detection limits [37]. Interestingly, very few studies compare the analytical performances of HPLC and MEKC or other separation techniques for the determination of potentially toxic phytochemicals found at low levels in our food chain [37,38,39,40].

Here, we developed and compared for the first time HPLC and MEKC methods for the determination of food flavouring ingredients. The reversed-phase HPLC method was developed by evaluation of three commercial columns and optimisation of the gradient conditions. MEKC with in-line sample concentration (stacking) method was developed by evaluation of natural bile salt micelles and optimisation of sample and background solution (BGS) conditions. The analytical performance of both methods was determined. Methanol (MeOH) extraction, which has been shown to be an efficient and simple sample preparation technique for alkenylbenzenes [33] was then optimised to obtain the best recovery for actual samples. Real sample application of the selected analytical method was then demonstrated in the analysis of real food flavouring samples.

## 2. Material and Methods

### 2.1. Standards and Reagents

Chemicals used in this study (disodium hydrogen orthophosphate anhydrous, estragole, eugenol, methyleugenol, myristicin, n-nonyl-β-d-glycopyranoside safrole, sodium cholate, SDS, sodium phosphate monobasic dihydrate and trifluoroacetic acid (TFA)) were obtained from Sigma-Aldrich (Castle Hill, New South Wales, Australia). Purified water was obtained from a Milli-Q system (Millipore, MA, USA). The organic solvents acetonitrile (ACN), acetone, ethanol (EtOH) and methanol (MeOH) were HPLC or analytical grade and obtained from Sigma-Aldrich (St. Louis, MO, USA).

For HPLC analysis, mobile phases were prepared by adding appropriate volumes of TFA to purified water or ACN. Prior to use, mobile phases were sonicated and filtered with 0.45 µm filter (Millipore, MA, USA). Each stock standard solution (5 mL) was prepared by mixing 5 µL of the analyte with 4.995 mL of 80% MeOH. The solutions were sonicated and stored at 2–8 °C. The resulting concentrations were 1.061 mg/mL (eugenol), 1.040 mg/mL (methyleugenol), 1.140 mg/mL (myristicin), 0.970 mg/mL (estragole), and 1.101 mg/mL (safrole). Standard solutions for injection were prepared by mixing appropriate volumes of the stock solution and 50% MeOH.

For MEKC analysis, stock solutions of 200 mM phosphate buffer (pH 11), 400 mM sodium cholate and 0.1 M NaOH were prepared in purified water. Phosphate buffer was prepared by mixing appropriate amounts of disodium hydrogen orthophosphate anhydrous and sodium phosphate monobasic dihydrate in purified water. The pH of phosphate buffer was adjusted using 0.1 M NaOH. Prior to use, all solutions were sonicated and filtered with a 0.45 µm disposable nylon filter. Background solution (BGS) were prepared by mixing the phosphate buffer, sodium cholate, acetone, and purified water at appropriate volumes. Stock solutions of analytes were prepared in 15 mM SDS and stored at 2–8 °C.

### 2.2. Instrumentation and Software

Reversed-phase HPLC was performed using a Dionex HPLC system, consisting of Ultimate 300 pump, Ultimate 300 Colum Compartment and Ultimate 3000 diode array detector and an Ultimate 300 autosampler (Sunnyvale, CA, USA). A GEMINI C18 (150 mm × 2.4 mm i.d., 5 µm particle size) from Phenomenex (Lane Cove West, NSW, Australia) was used as chromatographic column. Data acquisition was performed using Chromeleon 7.2.7 software (Sunnyvale, CA, USA). MEKC was conducted using the Agilent 3-D CE system (Agilent, Santa Clara, CA, USA), with 50 µm inner diameter and 375 µm outer diameter fused-silica capillary (Polymicro, Phoenix, AZ, USA). The effective capillary length was 29 cm (total length was 37.5 cm).

### 2.3. Sample Preparation

A 1 g of sauce or 0.2 g of dry plant material (e.g., leaf) sample was weighed in a 20 mL glass vial. 10 mL methanol was added to the sample and the mixture was sonicated for 15 min. A few ice cubes were added to the sonicator bath to maintain the water at room temperature. Then, 1 mL of the methanol extract was centrifuged for 5 min at 1000 rpm. The supernatant was filtered through a 0.45 µm nylon filter and then diluted 1:1 with purified water prior to HPLC analysis.

### 2.4. Reversed-Phase HPLC Conditions

The compounds were separated using a gradient mobile phase consisting of 0.1% (*v*/*v*) TFA in purified water (solvent A) and 0.1% (*v*/*v*) TFA in ACN (solvent B). Gradient conditions were: 0.0–1.0 min, 50% B; 1.0–13.0 min, 50–70% B; 13.0–14.0 min, 70% B; 14.0–15.0 min, 70–50% B; and 15.0–20.0 min, 50% B. Flow rate was 1.0 mL/min. The injection volume was 20 µL. The column temperature was maintained at 25 °C. The detection wavelength was set at 280 nm.

### 2.5. MEKC Analysis

A new capillary was conditioned with purified water, 0.1 M NaOH, purified water and BGS for 10, 30, 10, and 30 min, respectively. At the beginning of each testing day, the capillary was flushed with purified water for 5 min, 0.1 M NaOH for 10 min, purified water for 5 min and BGS for 10 min. In between runs, the capillary was flushed with purified water, 0.1 M NaOH, purified water and BGS for 1, 3, 1, 6 min, respectively. The detection wavelength was set at 200 nm. The capillary temperature was maintained at 20 °C. Stacking injection was by pressure at 50 mbar for 25 s.

## 3. Results and Discussion

### 3.1. Reversed-Phase HPLC Method Development

**Figure 1:**
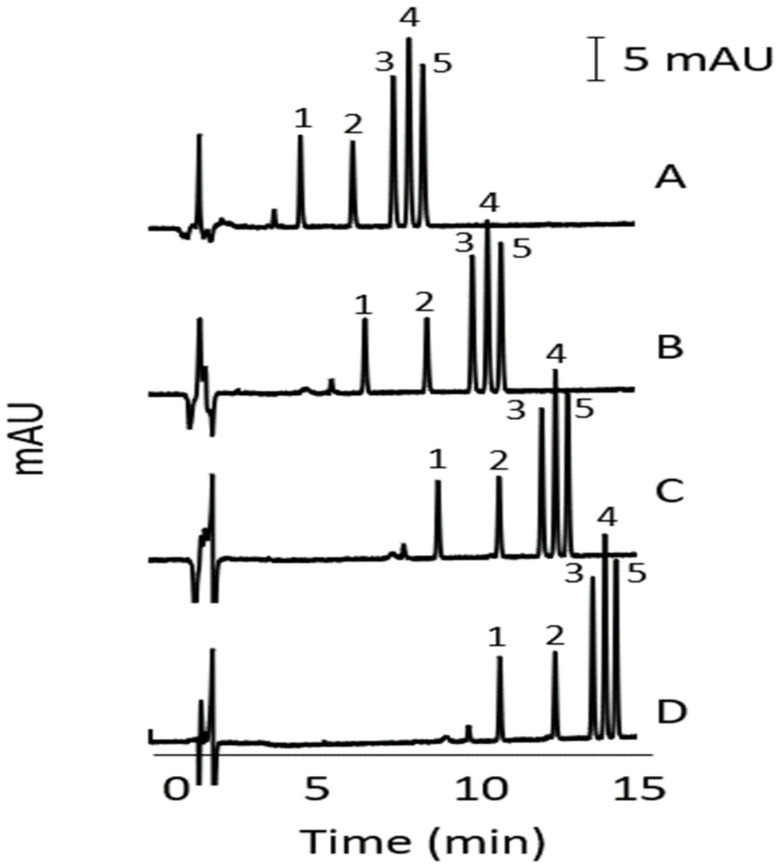
HPLC of alkenylbenzenes using different gradient conditions. % ACN in the initial mobile phase was 50%B (**A**), 40%B (**B**), 30% B (**C**), and 20% B (**D**). Other conditions are described in Section 2.4 and Section 3.1. Peak identity: eugenol (1), methyleugenol (2), myristicin (3), safrole (4), and estragole (5). Concentration of analytes (µg/mL): eugenol (3.5), methyleugenol (0.9), myristicin (20.3), safrole (8.5), and estragole (18.2).

The commercial analytical columns with the manufacturer’s recommended flow rate and injection volume used in this study are summarised in Table 2. Separations were evaluated using gradient elution with increasing concentrations of can in the mobile phase (increasing % of mobile phase B). The results indicated better separation performance with the Phenomenex GEMINI C18 column and thus the gradient conditions for this column were further optimised. Figure 1 shows the effect of the % ACN in the initial mobile phase on the separation of the alkenylbenzenes. The gradient conditions were: 0.0–1.0 min, % B (20%, 30%, 40%, 50%); 1.0–13.0 min, 70% B; 13.0–14.0 min, 70% B; 14.0–15.0 min, % B (20%, 30%, 40%, 50%). The column was conditioned with the initial mobile phase condition for 5 min prior to each sample injection. The % ACN at the start of the run in Figure 1A–D was 50%, 40%, 30%, and 20%, respectively. The separations were similar with all the conditions used but faster analysis was achieved with 50% © at the start of the run. All analytes were successfully separated. The identification of the peaks was performed by running individual analytes and by increasing the concentration of one analyte at a time in the standards mixture. The gradient condition in Figure 1A was then chosen with a run time (including conditioning) of 15 min. Compared to other reversed-phase HPLC methods reported in the literature [33], our method was faster and able to separate more alkenylbenzenes.

### 3.2. Stacking-MEKC Method Development

**Figure 2:**
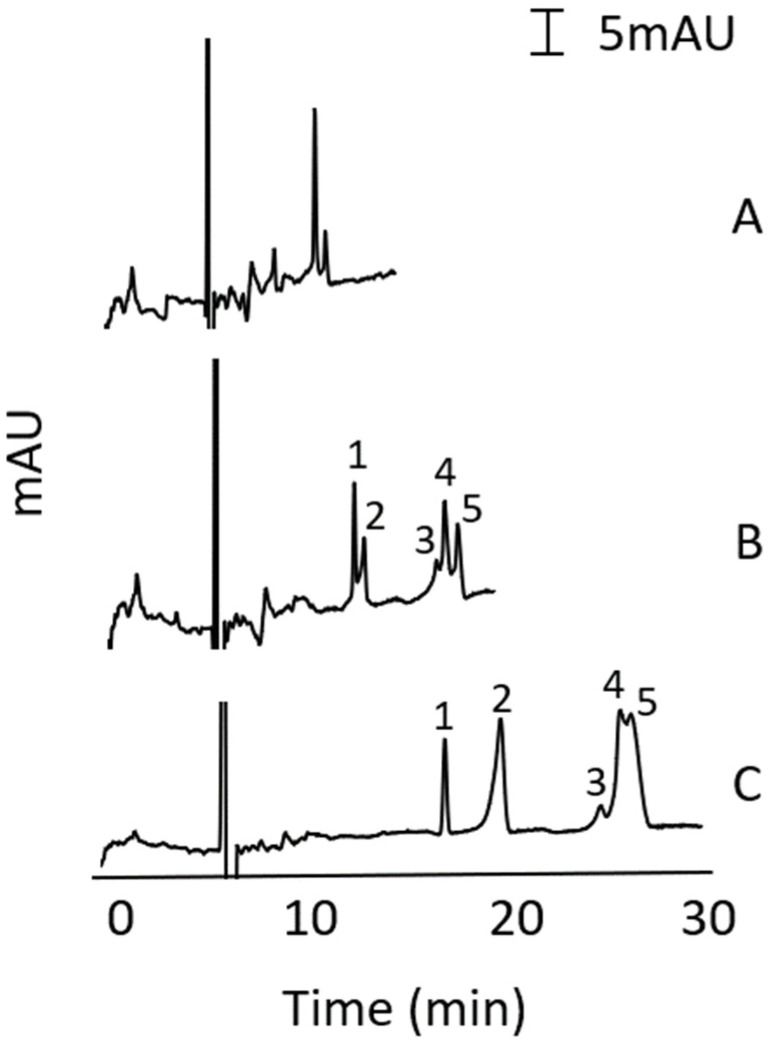
Effect of sodium cholate concentration in the MEKC separation of five alkenylbenzenes. BGS was 25 mM (**A**), 50 mM (**B**), and 100 mM (**C**) sodium cholate and 100 mM sodium phosphate buffer (pH 11). Injection was by pressure at 25mbar for 5s. Other conditions were described in Section 2.5. Peak identity: eugenol (1), methyleugenol (2), myristicin (3), estragole (4) and safrole (5). Concentration of analytes (µg/mL): eugenol (4.6), methyleugenol (3.6), myristicin (31.4), safrole (17.7), and estragole (18.2).

MEKC separations using SDS micelles in acidic and basic media and from various bile salts in neutral to basic media were investigated. Note that bile salts are only soluble in neutral to basic media. The most promising results were obtained using a BGS containing sodium cholate in 100 mM sodium phosphate buffer (pH 11), thus the BGS with sodium cholate in the pH 11 buffer was further optimised. Figure 2 shows the effect of sodium cholate concentration (25, 50, 100 mM) on the MEKC separation of the alkenylbenzenes at pH 11. With 100 mM sodium cholate in the BGS (see Figure 2C), compete separation of eugenol and methyleugenol (peaks 1 and 2) was achieved, but there was only partial separation of myristicin, estragole, and safrole (peaks 3, 4 and 5).

**Figure 3:**
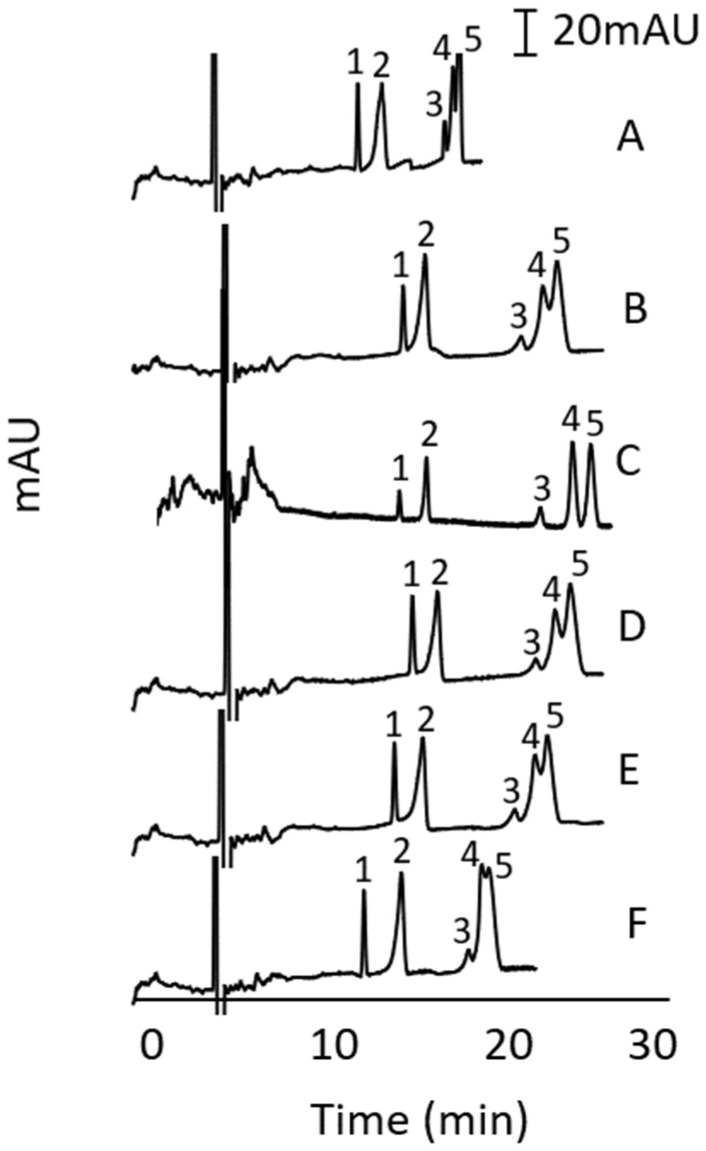
Effect of different additives on the MEKC of alkenylbenzenes. BGS was 10 mM n-nonyl-β-d-glycopyranoside (**A**), 3% methanol (**B**), 3.5% acetone (**C**), 3% ethanol (**D**), 3% acetonitrile (**E**), and no additive (**F**), 100 mM sodium cholate and 100 mM phosphate buffer (pH 11). Concentration of analytes (µg/mL): eugenol (7.9), methyleugenol (8.7), myristicin (35.8), safrole (26.4), and estragole (18.2). Other conditions and peak identity were the same as in Figure 2.

Various additives to the BGS containing 100 mM sodium cholate and 100 mM sodium phosphate buffer (pH 11) were then studied to improve the MEKC separation. The additives were a non-ionic surfactant (n-nonyl-β-d-glycopyranoside) and various organics solvents including MeOH, acetone, EtOH and ACN at different concentrations. The best result for each additive is shown in Figure 3 using 10 mM non-ionic surfactant (A), 3% MeOH (B), 3.5% acetone (C), 3% EtOH (D) and 3% ACN (E). Among the conditions presented in Figure 3, the separation of all peaks was obtained with 3.5% acetone (see Figure 3C) in the BGS.

### 3.3. Stacking Method Development in MEKC

**Figure 4:**
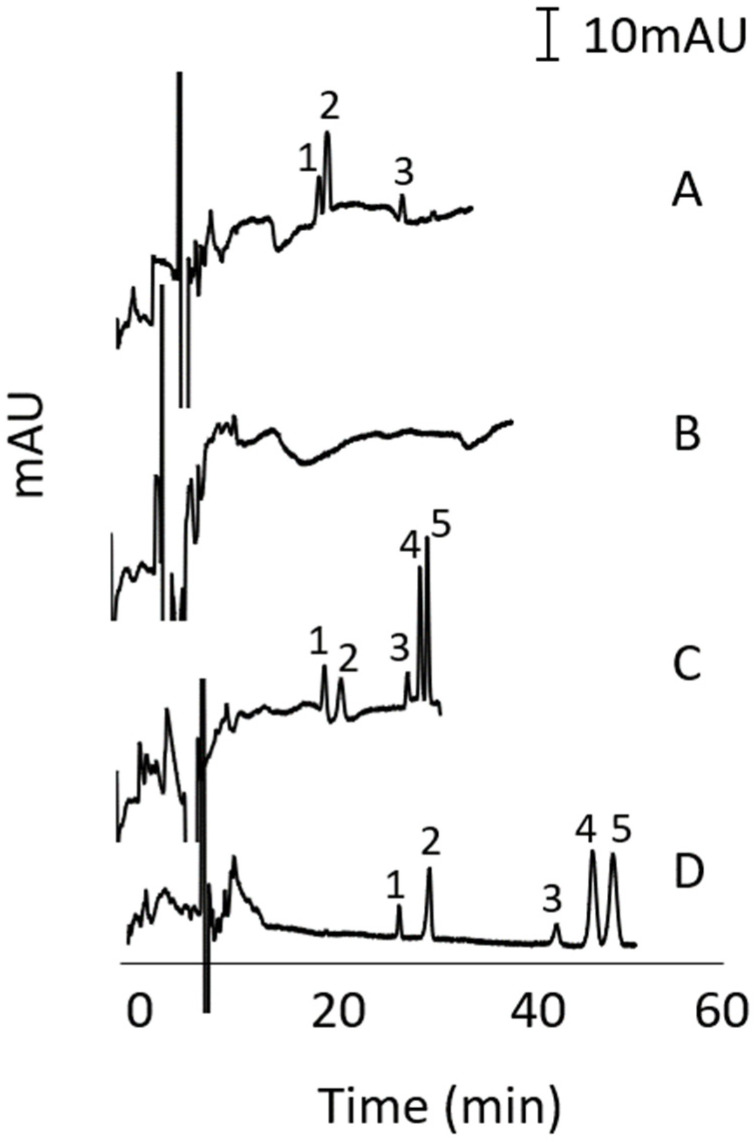
Effect of different sample diluents in the stacking-MEKC of alkenylbenzenes. Sample diluents were 10% MeOH in phosphate buffer (**A**), 10% MeOH in water (**B**), 15 mM SDS in water (**C**), and no diluent (**D**) in 3.5% acetone with 100 mM sodium cholate and 100 mM phosphate buffer (pH 11). Stacking injection was by pressure at 50 mbar for 25 s in (**A**–**C**). Typical injection was by pressure at 25 mbar for 5 s in (**D**). Concentration of analytes (µg/mL) in (**D**): eugenol (7.9), methyleugenol (8.7), myristicin (35.8), safrole (26.4), and estragole (18.2). Concentration of analytes (µg/mL) in (**A**–**C**): 1/10 of the concentrations in (**D**). Other conditions and peak identity were the same as in Figure 2.

Using the conditions described for Figure 3C, different stacking conditions were tested to increase the peak heights of the analytes without compromise to the separation performance. To evaluate the stacking, the sample solutions prepared in different diluents were injected as a long plug (50 mbar for 25 s) (see Figure 4A–C). The typical injection which was 1/10 × shorter than the stacking injection is shown in Figure 4D for comparison. The concentration of the analytes in the stacking injections were 10 × lower than in the typical injection. The sample diluent in Figure 4A was 10% MeOH in 100 mM sodium phosphate buffer (pH 11) to induce stacking via sweeping [41,42]. The sample diluent in Figure 4B was 10% MeOH in purified water and in Figure 4C was 15 mM SDS in purified water to produce stacking by field enhancement [43] and field enhancement with SDS micelles [44], respectively. Note that the CMC of SDS in water is 8 mM. Among the tested stacking techniques, field enhancement with SDS micelles was found most effective (see Figure 4C).

The stacking via field enhancement with SDS micelles was caused by the change in the effective electrophoretic velocity of the analytes (that are solubilised by the SDS micelles) at the boundary between the sample and BGS zones. The effective electrophoretic velocity of the analytes in the sample zone was faster than that in the BGS zone. The sudden decrease in the effective electrophoretic velocity caused the enrichment of the analytes at the boundary. The sensitivity enhancement factor (SEF) for each analyte was calculated by dividing the peak height obtained in typical injection by the peak height obtained in stacking injection, then multiplied by the dilution factor (=10). For the results in Figure 4C, the SEF values for eugenol, methyleugenol, myristicin, estragole, and safrole were 11, 5, 13, 8, and 12, respectively. Longer than 25 s injection of 50 mbar caused the co-migration of the last three peaks. Thus, the optimum stacking-MEKC conditions are those described for Figure 4C.

### 3.4. HPLC and Stacking-MEKC Comparison of Analytical Figures of Merit

The analytical figures of merit obtained for the optimised HPLC (conditions in Figure 1A) and stacking-MEKC (conditions in Figure 4C) are summarised in Table 3.

#### 3.4.1. Linearity

The reversed-phase HPLC method showed a good relationship between the tested concentrations of standard solutions and the corresponding peak areas. The linear ranges were ~2 orders of concentration magnitude (see Table 3). The correlation coefficients (R^2^s) for the calibration graphs/lines were all >0.997, which were above the typically recommended requirement for R^2^ of >0.995, whereas in MEKC, the linear ranges using corrected peak area (peak area/migration time) for myristicin, safrole, and estragole were <1 order of concentration magnitude and the R^2^ for safrole was unacceptable or 0.972. Therefore, only the HPLC method passed the requirements of the linearity study.

#### 3.4.2. LOD and LOQ

The LOD and LOQ values were determined using a signal-to-noise (S/N) ratio of 3 and 10, respectively (see Table 3). The LOQ (S/N = 10) values were also verified as the lowest concentration in the determined linear ranges. The LOQ values in HPLC were in the range between 0.07 and 0.79 µg/mL. The LOQ values in stacking-MEKC were at least an order of magnitude higher compared to HPLC. The values in stacking-MEKC ranged between 1.5 and 7.1 µg/mL. In summary, the HPLC was more sensitive than the stacking-MEKC method.

#### 3.4.3. Intra- and Inter-Day Repeatability

The intra- and inter-day repeatability was assessed using two concentration levels (i.e., LOQ and 8 × LOQ). For intra-day repeatability, each concentration level was analysed in 10 replicates (n = 10), which were performed during a day. For inter-day repeatability, each concentration level was analysed in 5 replicates in a day, for 3 consecutive days (n = 15). The percentage RSD values obtained in HPLC and stacking-MEKC are summarised in Table 4. At the LOQ and 8 × LOQ, the HPLC method showed excellent repeatability for retention time with intra- and inter-day repeatability percentage RSDs that ranged from 0.1 to 0.4%. For peak areas in HPLC, the intra-day repeatability percentage RSDs ranged from 0.0 to 4.0%, while inter-day repeatability percentage RSDs ranged from 0.0 to 7.0%. The intra- and inter-day repeatability percentage RSDs for migration time in stacking-MEKC ranged from 1.3 to 5.2%. In stacking-MEKC, the intra-day repeatability % RSDs for corrected peak area ranged from 0.4 to 5.0%, while the values for inter-day ranged from 0.3 to 7.8%. In general, the HPLC was more repeatable than the stacking-MEKC method. Therefore, the HPLC method was used for the optimisation of the MeOH extraction method for food samples.

### 3.5. Optimisation of Sample Preparation via MeOH Extraction

The sample preparation via MeOH extraction was initially optimised using a commercial dried basil leaves sample. The dried basil leaves sample contained 3 of the 5 alkenylbenzenes (eugenol, methyleugenol and estragole). The extraction described by Gursale et al. [45] was modified and optimized to provide the best recovery. The effect of MeOH volume during extraction was first investigated. Briefly, a 0.2 g sample was extracted with 5, 10, 15, or 20 mL of MeOH. The sample was sonicated for 15 min and centrifuged for 5 min. The supernatant was filtered and then diluted 1:1 with purified water prior to HPLC analysis. Corrected peak area was calculated by dividing the peak area with the volume of MeOH used. The best extraction was obtained using 10 mM of MeOH, as it provided the highest corrected peak areas among all the volumes studied. The sonication times of 5, 10, 15, and 20 min were then studied. The HPLC peak areas for the analytes increased gradually when the sonication time was increased from 5 to 15 min, then was similar when the time was 15 and 20 min. The 15 min was then considered as the optimum sonication time.

### 3.6. Recovery

Recovery studies were conducted using two samples and by standard addition method. For myristicin and safrole, dried basil leaves sample were used as they only contain eugenol, methyleugenol, and estragole. For eugenol, methyleugenol and estragole, dried oregano leaves were used. The samples were spiked or fortified with known amounts of the alkenylbenzenes (7 concentration levels). Extraction of triplicate samples with 10 mL of MeOH, 15 min sonication time and sample dilution were carried out as described in Section 3.5. Percentage recovery values were calculated by dividing the found concentration by the nominal concentration, then multiplied by 100%. The nominal concentration was the sum of the analyte concentration in the sample and the added concentration. The results are summarized in Table 5, which shows the analyte concentration in the sample, added concentration, nominal concentration, found concentration, and percentage recovery values. The highest percentage recovery values of 69.4 to 91.1% were achieved with eugenol. The values for the other alkenylbenzenes ranged from 57.1 to 81.4%. These values seem to be suitable for the determination of alkenylbenzenes for application to food samples.

### 3.7. Method Application

**Figure 5:**
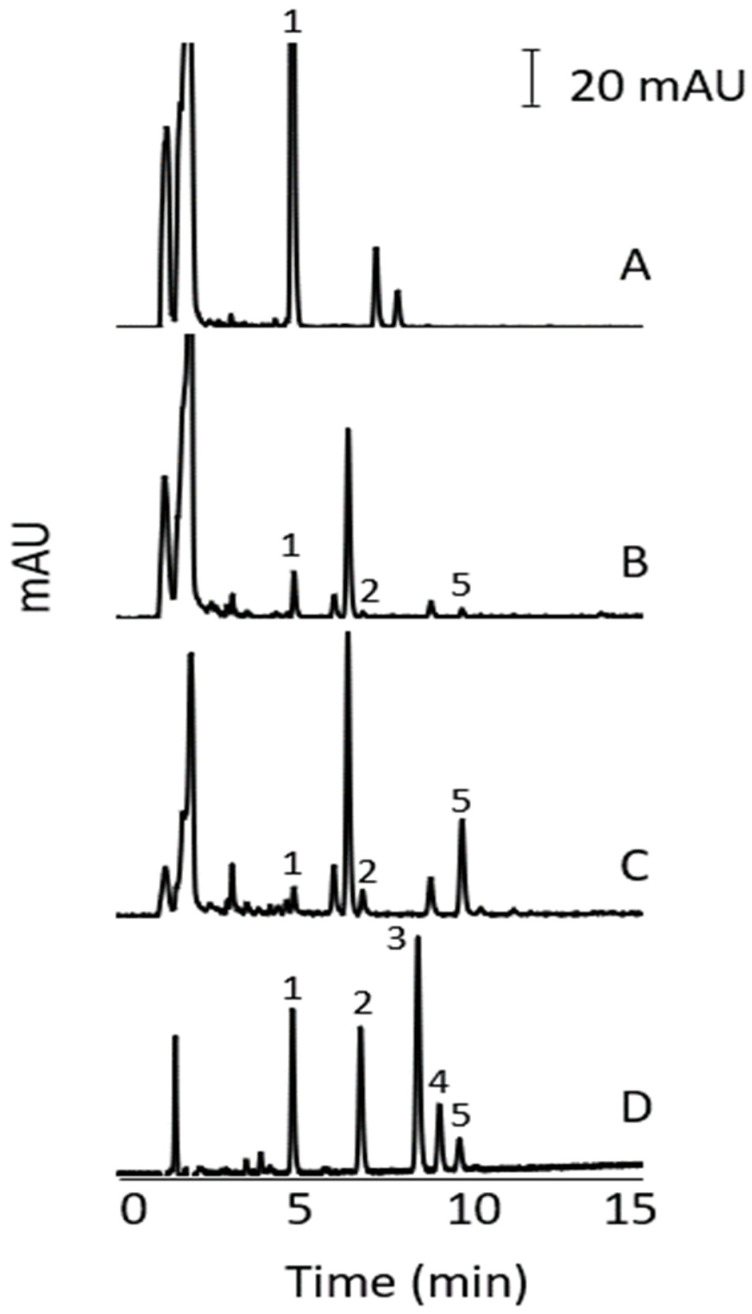
Representative HPLC chromatograms of cloves (**A**), Galiko basil sauce (**B**), basil leaves (**C**), and alkenylbenzenese standard solution (**D**). Experimental conditions were stated in Section 2.4 and peak identity as in Figure 1.

The optimised analytical method utilizing MeOH extraction and reversed-phase HPLC analysis was applied to the determination of the 5 alkenylbenzenes in fresh basil leaves, Galiko basil sauce (38% basil leaves), and cloves. The results obtained from three replicate analysis of each sample are summarised in Table 6. As expected, the highest eugenol content was found in cloves with 8.71 ± 0.09 mg/g, whereas methyleugenol and estragole were detected at the highest levels in basil leaves with 0.07 ± 0.01 and 0.72 ± 0.11 mg/g, respectively. A representative HPLC chromatogram for each sample is shown in Figure 5A–C. Figure 5D is HPLC of a standard mix for comparison. The targeted analytes seem to be well separated from the other components found in the tested real samples.

## 4. Conclusions

In the present work, reversed-phase HPLC and stacking-MEKC methods were developed for the determination of alkenylbenzenes in food flavouring ingredients. The analytical figures of merit determined for reversed-phase HPLC outperformed the stacking-MEKC method. The reversed-phase HPLC linear ranges were ~2 orders of concentration magnitude with correlation coefficients of >0.997 for the calibration graphs. The limit of quantitation (LOQ) values obtained for standard solutions were between 0.07 and 0.79 µg/mL. The intra- and inter-day repeatability obtained using standard solutions at the LOQ and 8 × LOQ for retention time and peak areas were <7.0%. Meanwhile, the optimised MeOH extraction method provided percentage recovery values from 57 to 91%. The analytical methodology that combined MeOH extraction and reversed-phase HPLC was then successfully applied to actual food flavouring ingredients. The analytical methodology will be implemented to actual food samples in the future. Using an ample number of real samples, we aim to determine the occurrence data and margin of exposure for the alkenylbenzenes. Therefore, we may be able provide guidance on the current potential health risks of these compounds that are ubiquitously found in our foods today.

## Figures and Tables

**Table 1:** Occurrence of eugenol, methyleugenol, myristicin, safrole, and estragole found in plants.

Plants	Alkenylbenzenes					Food
	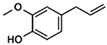 Eugenol	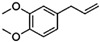 Methyleugenol	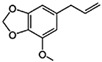 Myristicin	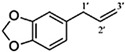 Safrole	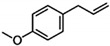 Estragole	
cloves	28.5 ± 0.4 mg/g [11]	Detected ^†^ [12]	Detected ^†^ [12]			various curries, jams, cooking rice
cinnamon	0.19–1.65 g/kg [13]		Detected ^†^ [12]			various curries, churros, donut, various pastries
nutmeg	0.32 mg/g [14]	8 mg/g [15]	280–420 mg/kg [16]	109.8 mg/mL of extract [17]		various curries, alfredo pasta, soufflés, beef stew, various baked products
sweet/holy basil, basil	540 mg/kg [11]	Detected ^†^ [18]	Detected ^†^ [12]		710 mg/kg [11]	various pesto sauces, Thai noodle and curry, caprese salad
star anise		98 mg/g [19]	Detected ^†^ [12]	66 mg/g [19]		Thai noodle, Chinese soups and stews
dill			28.1–76.3 mg/g [20]			various curries and soups, salad dressing, sandwich fillings
celery			Detected ^†^ [21]			chicken noodle soup, various stir fry dishes, various vegetarian dishes
ginger	Detected ^†^ [22]	14.0 ± 1.6 µg/g [23]		500 ± 36 mg/kg [24]		various teas, sushi, congee, various soups, various curries
tarragon	Detected ^†^ [25]	0.5–28.9% *v*/*v* [26]			17–75% *v*/*v* [26]	pasta, mojito, Béarnaise sauce
rosemary			Detected ^†^ [27]			various soups, salads, stews, and pasta sauces
thyme	0.021 mg/g [14]			detected^†^ [28]		seasoned roasted vegetables, various pasta sauces and soups, pizza toppings
bay leaves	110–120 mg/g [29]	90–120 mg/g [29]				beef stew, pate, various curries and soups
carrot		Detected ^†^ [30]	1.1–16.6 µg/g [31]			various soups, salads, noodle dishes, and curries, cakes, salads, coleslaw
pepper	11–120 mg/kg [32]	0.5–20.1 mg/kg [32]	0.2–6.1 mg/kg [32]	955 ± 80 mg/kg [24]	2.2–45.7 mg/kg [32]	various pasta and curry dishes, and sauces
fennel			Detected ^†^ [12]		2.0–3.0% *v*/*v* [26]	various pasta dishes, various salads and slaws, sausages

^†^ Detected but the actual amounts were not reported.

**Table 2:** Characteristics for the three tested commercial reversed-phase HPLC columns.

Column.	Specifications (Particle Size)	Flow Rate (mL/min)	Injection Volume (µL)
Thermo Fischer Hypersil GOLD C18	50 mm × 2.1 mm i.d. (3 µm)	0.2	5
Acclaim C18	100 mm × 2.1 mm i.d. (2.2 µm)	0.5	10
Phenomenex GEMINI C18	150 mm × 4.6 mm i.d. (5 µm)	1.0	20

**Table 3:** Linear regression data for calibration curves.

Method		Eugenol	Methyleugenol	Myristicin	Safrole	Estragole
HPLC	concentration range (µg/mL)	0.18–12	0.07–4.3	0.79–50	0.36–23	0.73–46
	slope of the line	251.27	1005.70	84.26	261.06	108.30
	intercept of the line	−0.0169	−0.0171	−0.0162	−0.0310	−0.0362
	correlation coefficient (R^2^)	0.9970	0.9967	0.9966	0.9968	0.9965
	LOD (µg/mL)	0.09	0.03	0.39	0.18	0.36
	LOQ (µg/mL)	0.18	0.07	0.79	0.36	0.73
stacking	concentration range (µg/mL)	1.6–50	1.5–24	3.7–29	7.1–21	6.0–37
MEKC	slope of the line	8590.2	6087.6	595.87	3020.2	1537.7
	intercept of the line	−13.801	+3.279	+0.374	−22.81	−10.36
	correlation coefficient (R^2^)	0.9972	0.9947	0.9964	0.9724	0.9975
	LOD (µg/mL)	0.41	0.38	1.8	2.7	4.3
	LOQ (µg/mL)	1.6	1.5	3.7	7.1	6.0

**Table 4:** Intra- and inter-day repeatability of HPLC and stacking-MEKC methods.

Method		Analyte	Concentration Levels (µg/mL)	% RSD (Retention Time ^1^/Migration Time ^2^)		% RSD (Peak Area/Corrected Peak Area ^5^)	
				Intra-Day (n = 10) ^3^	Inter-Day (n = 15) ^4^	Intra-Day (n = 10) ^3^	1nter-Day (n = 15) ^4^
HPLC	LOQ	Eugenol	0.2	0.2	0.4	4.0	3.3
		methyleugenol	0.1	0.1	0.4	3.8	3.9
		myristicin	0.8	0.2	0.5	0.0	0.0
		safrole	0.4	0.1	0.4	0.0	3.3
		estragole	0.7	0.1	0.4	3.2	1.7
	8 × LOQ	eugenol	1.4	0.4	0.3	0.7	1.1
		methyleugenol	5.4	0.4	0.3	0.5	0.9
		myristicin	6.3	0.3	0.3	0.0	0.1
		safrole	2.9	0.3	0.3	0.0	3.5
		estragole	5.8	0.3	0.3	0.5	7.0
stacking-MEKC	LOQ	eugenol	1.6	2.9	4.4	0.6	0.8
		methyleugenol	1.5	2.9	5.2	0.4	0.3
		myristicin	3.7	2.4	2.0	1.1	3.0
		safrole	7.1	1.5	1.4	3.4	3.6
		estragole	6.0	1.8	1.6	5.0	6.3
	8 × LOQ	eugenol	13.0	1.3	1.6	4.8	1.1
		methyleugenol	6.1	3.2	1.7	0.7	1.9
		myristicin	15.0	1.9	3.8	0.4	2.1
		safrole	11.0	1.4	1.9	4.4	6.1
		estragole	18.0	2.4	2.5	4.8	7.8

^1^ Retention time was used in HPLC. ^2^ Migration time was used in stacking-MEKC. ^3^ Each concentration level was replicated 10 times within a day. ^4^ Each concentration level was replicated 5 times within a day, for 3 consecutive days. ^5^ Corrected peak area was used in stacking-MEKC, corrected peak area = peak area/migration time. This was because of the different effective electrophoretic velocities of the analytes in MEKC.

**Table 5:** Percentage recovery study results in the MeOH extraction and reversed-phase HPLC analysis of the five alkenylbenzenes in fortified real samples.

Analyte	Sample Concentration (µg/mL)	Added Concentration (µg/mL)	Nominal Concentration (µg/mL)	Found Concentration (µg/mL)	% Recovery
eugenol	1.08	0.54	1.62	1.32 ± 0.00	81.5 ± 0.0
		1.08	2.16	1.79 ± 0.00	82.9 ± 0.0
		2.16	3.24	2.75 ± 0.01	84.9 ± 0.0
		4.32	5.40	4.92 ± 0.00	91.1 ± 0.0
		8.64	9.72	8.02 ± 0.01	82.5 ± 0.0
		17.28	18.36	16.19 ± 0.04	88.2 ± 0.0
		34.56	35.64	24.73 ± 0.06	69.4 ± 0.1
methyleugenol	0	0.21	0.21	0.12 ± 0.00	57.1 ± 0.0
		0.42	0.42	0.25 ± 0.00	59.5 ± 0.0
		0.84	0.84	0.56 ± 0.01	66.7 ± 0.0
		1.68	1.68	1.25 ± 0.00	74.4 ± 0.0
		3.36	3.36	2.46 ± 0.01	73.2 ± 0.0
		6.72	6.72	4.18 ± 0.04	62.2 ± 0.0
		13.44	13.44	7.72 ± 0.07	57.4 ± 0.1
myristicin	0	2.37	2.37	1.93 ± 0.00	81.4 ± 0.0
		4.74	4.74	2.98 ± 0.00	62.9 ± 0.0
		9.48	9.48	6.38 ± 0.00	67.3 ± 0.0
		18.96	18.96	12.72 ± 0.03	67.1 ± 0.0
		37.92	37.92	22.22 ± 0.01	58.6 ± 0.0
		75.84	75.84	47.66 ± 0.03	62.8 ± 0.0
		151.68	151.68	95.85 ± 0.05	63.2 ± 0.1
safrole	0	1.08	1.08	0.84 ± 0.00	77.8 ± 0.0
		2.16	2.16	1.38 ± 0.01	63.9 ± 0.0
		4.32	4.32	2.76 ± 0.00	63.9 ± 0.0
		8.64	8.64	5.63 ± 0.03	65.2 ± 0.0
		17.28	17.28	10.32 ± 0.03	59.7 ± 0.0
		34.56	34.56	23.37 ± 0.04	67.6 ± 0.0
		69.12	69.12	45.55 ± 0.07	65.9 ± 0.1
estragole	0.59	2.19	2.78	2.06 ± 0.00	74.1 ± 0.0
		4.38	4.97	3.83 ± 0.01	77.1 ± 0.0
		8.76	9.35	7.07 ± 0.01	75.6 ± 0.0
		17.52	18.11	14.00 ± 0.02	77.3 ± 0.0
		35.04	35.63	27.68 ± 0.02	77.7 ± 0.0
		70.08	70.67	56.14 ± 0.05	79.4 ± 0.1
		140.16	140.75	106.17 ± 0.06	75.4 ± 0.1

**Table 6:** Application of the optimized MeOH extraction and reversed-phase HPLC to basil leaves, commercial basil sauce and clove samples.

Sample	Amount Found (mg/g)				
	Eugenol	Methyleugenol	Myristicin	Safrole	Estragole
basil leaves	0.27 ± 0.01	0.07 ± 0.01	ND	ND	0.72 ± 0.11
Galiko basil sauce	0.12 ± 0.01	0.004 ± 0.00	ND	ND	0.07 ± 0.01
cloves	8.71 ± 0.09	ND	ND	ND	ND

ND: not detected.

## Sample Availability

Samples of the compounds including eugenol, methyleugenol, myristicin, safrole and estragole are available from the authors.

## Abbreviations

HPLC	High performance liquid chromatography
MEKC	Micellar electrokinetic chromatography
GC	Gas chromatography
